# Changes in Immune Response during Pig Gestation with a Focus on Cytokines

**DOI:** 10.3390/vetsci11010050

**Published:** 2024-01-22

**Authors:** Carolina Velez, Delia Williamson, Mariela Lorena Cánovas, Laura Romina Giai, Catrin Rutland, William Pérez, Claudio Gustavo Barbeito

**Affiliations:** 1 Laboratory of Histology, Faculty of Veterinary Science, National University of La Pampa (UNLPam), Santa Rosa 6300, Argentina; cvelez@vet.unlpam.edu.ar (C.V.); dwilliamson@vet.unlpam.edu.ar (D.W.); romigiai@gmail.com (L.R.G.); 2 National Scientific and Technical Research Council of Argentina (CONICET), Buenos Aires 2690, Argentina; barbeito@fcv.unlp.edu.ar; 3 Sutton Bonington Campus, Faculty of Medicine and Health Sciences, University of Nottingham, Nottingham NG7 2RD, UK; 4 Department of Veterinary Anatomy, University of Montevideo, Montevideo 11600, Uruguay; 5 Laboratory of Descriptive, Comparative and Experimental Histology and Embriology (LHYEDEC), Department of Basic Sciences, Faculty of Veterinary Science, National University of La Plata (UNLP), La Plata 1900, Argentina

**Keywords:** epitheliochorial placenta, embryo death, cytokines, innate immunity, asymmetric antibodies

## Abstract

**Simple Summary:**

Immunity during pig pregnancy has been especially studied during the periimplantation period, in which there is an important relationship between sex steroids and cytokines. Although part of the abundant embryonic/fetal deaths in pigs occur in more advanced periods of gestation, there are few studies that analyze these stages. This review analyzes the changes in the immune response, emphasizing the local response and cytokines, throughout pig gestation. The very great variations that occur throughout the periimplantation period and the complexity of the response at the time of placental remodeling (60–70 dpc) when increase anti and proinflammatory cytokines.

**Abstract:**

Pigs have the highest percentage of embryonic death not associated with specific diseases of all livestock species, at 20–45%. During gestation processes, a series of complex alterations can arise, including embryonic migration and elongation, maternal immunological recognition of pregnancy, and embryonic competition for implantation sites and subsequent nutrition requirements and development. Immune cells and cytokines act as mediators between other molecules in highly complex interactions between various cell types. However, other non-immune cells, such as trophoblast cells, are important in immune pregnancy regulation. Numerous studies have shed light on the crucial roles of several cytokines that regulate the inflammatory processes that characterize the interface between the fetus and the mother throughout normal porcine gestation, but most of these reports are limited to the implantational and peri-implantational periods. Increase in some proinflammatory cytokines have been found in other gestational periods, such as placental remodeling. Porcine immune changes during delivery have not been studied as deeply as in other species. This review details some of the immune system cells actively involved in the fetomaternal interface during porcine gestation, as well as the principal cells, cytokines, and molecules, such as antibodies, that play crucial roles in sow pregnancy, both in early and mid-to-late gestation.

## 1. Introduction

Embryonic death occurs in all eutherian mammals, both as a consequence of injurious agents and due to unknown factors. Some species, such as the European hare (*Lepus europeus*), a lagomorph, and the plain viscacha (*Lagostomus maximus*), a rodent and the only living species within the genus *Lagostomus*, have very high rates of embryonic death under physiological conditions [1,2,3] and, therefore, they have been proposed as models to study this process. Among livestock species, the pig has one of the highest percentages of embryonic death; between 20 and 45% of prenatal loss is not associated with specific diseases. The maximum values of loss have been identified at the peri-implantation stage and between days 50 and 90 of a 114-day gestation. The contributing factors identified to date include poor vascularization, an imbalance between the growth of the conceptus (embryo or fetus and its annexes) and the uterus, and alterations in the local immune response [4]. At a global level, pig farming is a primary livestock activity representing 33.8% of total meat production, making it an important source of high-quality animal protein for human consumption [5]. Without a doubt, reproductive demands in the search to produce more offspring per litter have contributed to the abundant loss of embryos.

In the placenta of eutherian mammals there are multiple tissue antigens that differ between the fetus and the mother, and there are potentially recognizable tissue alloantigens within the maternal immune system. This raises questions as to why maternal immune cells do not induce a response against these alloantigens, and how does the mother protect herself against microbial agents without triggering a lethal immune response against fetal tissues [6] For this reason, various research efforts have focused on studying the mechanisms responsible for preventing rejection and enabling harmonious coexistence between the embryo and the mother. Throughout the 20th century and based on the hypotheses generated by Peter Medawar [7] in the 1950s, different models have been developed to explain the immune particularities that occur in the pregnant uterus and to understand the maintenance of these alloantigens [8]. Subsequently, Tom Wegmann [9] and other researchers postulated that, during the majority of each pregnancy, Th2 cytokines are predominant, inhibiting the inflammatory response and cellular immunity. This paradigm, at the beginning of this century, was seen as an oversimplification, and it has been made more complex with the incorporation of a wide variety of cells and cytokines. It has been suggested that Tr1 lymphocytes and Th3 cytokines (TGF beta) work together, with Th2 cytokine regulating the maternal immune response towards embryonic tolerance. This implies a Th1/Th2/Th3/Tr1 regulatory system during pregnancy [10], and in this new paradigm other cell types such as Th17 cells have been included. However, it is of interest that we have not found references to these cells during porcine gestation.

During pregnancy, both the maternal innate and acquired immune systems are involved. Particularly noteworthy are the actions of natural killer (NK) cells, macrophages, lymphocytes such as T and B cells, dendritic cells, diverse cytokines, and other soluble molecules that participate in a coordinated manner to carry the pregnancy to term [11]. However, a characteristic of the immune response at the maternal–fetal interface is that non-immune cells such as trophoblastic cells and endometrial fibroblasts produce substances important in immunomodulation [12].

It is currently considered that, in mammals, implantation requires endometrial inflammation; in eutherians, there is a long period of tolerance that enables their gestation periods to be longer than those of marsupials [13]. This change has been related to an antiestrogenic action and the consequent stimulation of the progesterone pathway. It has been found that a pleiotropic transcription factor, Hand-2, is expressed in the post-implantation period in eutherians but not in marsupials. This expression occurs even in non-invasive placentas without decidual reactions including that of the pig [14]. The action of steroid hormones is essential. Estrogens predominate before implantation and induce the inflammatory response; then, the predominance of progesterone favors an anti-inflammatory response during the majority of the pregnancy, and at the end of pregnancy estrogen concentrations rise again. Despite the great differences between the placentas and pregnancies of different eutherian species, the model presented previously is quite well conserved. The main cellular and molecular actors that play important roles during pregnancy are described below, and the data known specifically for pig pregnancy are presented.

## 2. Immune Cells in the Pig Placenta

The concentration of immune cells in the endometrium varies both throughout the estrous cycle and throughout pregnancy. In both cases, the most abundant cells are T lymphocytes and NK cells. In the case of the estrous cycle, a large increase occurs in the proliferative phase, regulated by the microbiota of the uterine lumen [15]. In the early stage of gestation, when intimate contact between fetal and maternal tissues is established, the concentration of maternal uterine lymphocytes decreases in the luminal epithelium and increases in the endometrial stroma at conceptus attachment sites, and it is the conceptus that influences the distribution of T and NK lymphocytes during early pregnancy [16,17,18,19]. In addition, the number of leukocytes is three times higher in the endometrium at the implantation sites than in the tissue between them. These leukocytes also mainly consist of T cells and NK cells [16]. In pigs, increases in the mRNA expression of the chemokines CXCL9, CXCL10, and CXCL11, and their receptor CXCR3 mRNAs, have been found to be greatest on day 15 of pregnancy but to decrease thereafter. The expression of these chemokines is positively regulated by IFN-γ and acts by attracting different CD4 and CD8 T lymphocytes and NK cells, which increase in number during this stage of pregnancy and decrease in the later stages [20].

### 2.1. NK and T-Cells

A fundamental event during the early evolution of the eutherian placenta was the appearance of a different variety of NK cells: uterine natural killer (uNK) cells. These cells, with functions that differ from the NK cells in the rest of the organism, are essential for regulating the remodeling of uterine spiral arteries in rodents and primates, such as monkeys and humans [21]. These uNKs cells seem to have emerged at the beginning of the evolution of eutherian placentation and have been studied in human, mouse [22], and other rodent placentas (such as *Lagostomus. maximus*) [23]. The analysis of various species such as cattle, sheep, and pigs, which have non-invasive placentas [16,24,25], has demonstrated that NK cells are abundant in the endometrium, particularly at sites of trophoblast adhesion, as well as in the stroma and endometrial glands. Engelhardt and King [11] proposed that these cells are similar to the uNK cells observed in rodents and primates. However, these cells lack characteristics present in human and rodent uNK cells, such as abundant large cytoplasmatic granules, and are more similar to non-uterine NK cells. Some authors propose that species that exhibit epitheliochorial placentation do not have true uNK cells [26], although other authors [27] do name them uNK cells. Rodriguez-Martinez [28] describes the NK cells in pigs as small agranular lymphocytes, and we have not identified any subsequent works that use the term uNK when comparing the NK cells found in the porcine endometrium with the uNK cells within primates and rodents. Engelhardt et al. [17] proposed that the production of INF-ɣ by NK cells in the uterus is crucial for the success of angiogenesis and placental neovascularization in sows, and therefore plays a fundamental role in the development of a successful placenta and embryonic survival.

NK cells change in their density and location depending on the implantation phase. Prior to implantation, they mainly reside in the subepithelial endometrial connective tissue. However, in more advanced stages of implantation, the numbers decrease in this location and concomitantly increase in the glandular layer, suggesting a relocation induced by implantation. All of the NK cells identified in porcine uterine in one study were CD56(+), although some were also CD3(−) CD8(+), and others were CD16(+); however, the use of an unspecific porcine antibody in this study relativizes these results. NK cell numbers have also been shown to decrease significantly once the definitive placenta has been established, before increasing again at the end of gestation. However, no significant differences between the number of circulating NK cells in both pregnant and non-pregnant pigs have been observed [18]. More recently, Stas et al. [29] found that NK cells expressed perforin (likely with cytolytic function) in the placenta 100 days into gestation. NK cells also increase at the maternal–fetal interface during porcine reproductive and respiratory syndrome virus infection [30]. This increase has been associated with the trophoblastic damage occurring following the contraction of this disease [31].

In summary, there are NK cells in the porcine uterus with some important immunoregulation functions. In pigs, these cells do not have the same morphological and histochemical characteristics found in human and rodent uNK cells, such as large +PAS granules recognizable by DBA lectin and its very large and euchromatic nuclei. The morphological characteristics of pig uterine NK cells are very similar to NK cells in other anatomical locations.

Different populations of T cells are found in the uterus during pig gestation. The classic Th1/Th2 paradigm that prevailed to explain the immune regulation of pregnancy at the end of the 20th century proved to be a simplification when other variants of regulator T lymphocytes (Treg) with functions in the regulation of pregnancy began to be found. These cells have been studied in detail in humans, and among them are Th3 Treg; producers of immunomodulatory cytokines such as transforming growth factor ß1 (TGFß1), IL-10, and IL-4; and Tr1 Treg, which also produces TGFß1 and IL-10, but not IL-4 [32]. Th3 cells have been found [33] in other localization in pigs, but references to their existence in the uterus or placenta have not been found. Thus, Th3 cytokines such as TGFß1 must be produced by other cells during pig pregnancy.

Lymphocytes are 15% leukocytes in the pre-attachment stage, increasing to 54% after attachment at gd15. This percentage is at its maximum (85%) at gd 30 after the formation of the placenta. T-cell populations are found in the maternal placenta at advanced gestation (100 dg), while naïve lymphocytes are predominant in the fetal placenta [34]. These results are variable in relation to the lymphocyte population, but with respect to localization, T-cell subpopulations are found in comparable proportions at and between embryonic attachment sites. However, circulating lymphocyte numbers do not change during the peri-implantational period or in the placentation stage [35]. In the peri-implantational stage, the numbers of T helper, Treg, Tγδ and T cytotoxic cells increase at day 13 of gestation, but not at day 6 [36]. The Tγδ cells found have been classified into two subpopulations: CD2^+^ and CD2^−^. Some of these cells express perforin [29]. CD4 and CD8 T lymphocytes of the maternal embryo/fetal interface, as observed in NK cells, increase during intracellular infections, such as porcine reproductive and respiratory syndrome virus, and CD8 effector cells may contribute to trophoblast damage, as could NK cells [37]. This recent study did not report the existence of subpopulations of placental Tregs in pig, as was found in other species.

### 2.2. Polymorphonuclear Neutrophils (PMNNs), Dendritic Cells, and Other Cells

It has been shown that pig seminal plasma stimulates the arrival of PMNNs to the uterus [28]. PMNNs remove excess spermatozoa through phagocytosis in many mammals (including swine) and maintain a microenvironment favorable for fertilized oocyte implantation and further embryo development [38].

Dendritic cells have been studied in great depth in hemochorial placentas, where they are considered fundamental for the initiation of the pregnancy-specific immune response, but have not been studied in such detail in pigs. Linton et al. [39] demonstrated their presence in the pregnant and non-pregnant porcine uterus, although they found differences between the two conditions and determined that dendritic cells had the capacity to produce angiogenic factors and express toll-like receptors. It has also been shown that a decrease in number of dendritic cells accompanies the decrease in angiogenesis that occurs in early- and mid-pregnancy developmental arrest and embryonic loss [40].

Although an increase in the number of other cell types such as mast cells, eosinophils, and macrophages has been described during the implantation period [41], in-depth studies of these cells have not been conducted. For example, we have not found reports relating to the M1-M2 differentiation processes in macrophages in pigs, yet this has been described in depth in other species [42].

## 3. Cytokines in the Pig Placenta

Numerous studies have revealed that cytokines play a crucial role in regulating maternal immune tolerance towards the implanted conceptus, as well as in the changes that occur in endometrial architecture to promote uterine receptivity and vascular remodeling to facilitate the exchange of nutrients and waste between the mother and the fetus [43,44,45,46].

Specifically, numerous studies on mammals have described the role of other cytokines that may regulate the immune environment throughout porcine gestation [34,47,48,49]. In our laboratory, we have investigated the expression of several cytokines during porcine placentation, including INFγ, interleukin-1β (IL-1β), IL-2, IL-4, IL-6, IL-10, IL-12, IL-15, and IL-18, aiming to elucidate their roles at the fetal–maternal interface, their effects on immune cells, and how they modulate different stages of porcine placentation [50,51,52]. It is noteworthy that during the stages of extensive placental remodeling at 30 and 70 days of gestation (dg), high levels of proinflammatory cytokines were observed in placental tissue, including IL-4, suggesting that they might regulate an immune environment otherwise detrimental to the success of gestation. Towards the end of gestation, the concentration of these cytokines in the placenta decreases significantly, while their levels increase in the serum, possibly preparing the body for labor and placental expulsion [51,52]. In Table 1, we can see a description of the different cytokines in pigs, and in Table 2 we can see the cytokines in relation to different gestational periods and their principal events.

## 4. Interleukins

### 4.1. Interleukin 1 β (IL-1β)

IL-1β is a proinflammatory cytokine that acts as a central mediator of inflammation and innate immunity in mammals [76,77]. IL-1β influences conceptus implantation, invasion, and fetomaternal immunotolerance [78,79]. It also plays a crucial role in the dialogue established between the uterus and the conceptus during implantation in humans, mice, and pigs [53,54,56,80,81,82]. Tuo and Bazer [53] were the first to demonstrate that porcine *conceptus* produces IL-1β in differential forms during early development and elongation. IL-1β expression in the conceptus rapidly increases during the short elongation period and then decreases dramatically (2000 times) as the conceptus attaches to the uterine surface, but then becomes nearly undetectable by day 14 of gestation [54].

Positive interactions between estrogen receptor (ER) signaling pathways and IL-1β have been reported in reproductive tissues, particularly in human endometrial epithelial cell lines, suggesting that estrogens and IL-1β may collaborate to promote implantation [83]. In pigs, it has been suggested that IL-1β is necessary for promoting early *conceptus* development and rapid elongation, enhancing uterine receptivity for implantation, and increasing the permeability of endometrial blood vessels, thereby facilitating fetal–maternal hemotrophic exchange [54,55,56]. The *conceptus*’s production of a unique interleukin 1β, IL1 β2, temporally increases during the period of trophoblast remodeling during elongation in the first 14 dg. The inhibition of these cytokines generates a failure in conceptus elongation [58]. The role of IL-1β during the implantation period is well known, particularly in pigs, as numerous studies have clarified its involvement in trophoblast development during early gestation and have shown how it communicates with the endometrium, thus enabling processes necessary for proper implantation [54,56]. IL-1β is also produced in the trophoblast of the 12 dg *conceptus* [59], and is recognized by the endometrial epithelium receptor activating different mechanisms related to implantation [57].

Despite this early gestation knowledge relating to IL-1β, few studies have examined the physiological levels of IL-1β in sows during the rest of gestation. Regarding local placental levels of IL-1β during porcine gestation, Tuo and Bazer [53] found mRNA expression in conceptus and fetal placenta from days 11 to 45, after which levels significantly decreased. However, in our laboratory, IL-1β exhibited a peak in concentration within the fetal placenta at day 60 and in the maternal placenta at day 70, followed by a decrease by day 114 [51].

The period between 60 and 70 days represents a critical phase in porcine gestation. At 60 days, the placenta completes its exponential growth, generating secondary and tertiary villi to support the weight of the fetuses, which then also enter their own exponential growth phase. Furthermore, between 60 and 70 days, the most significant placental remodeling of the pregnancy takes place [84].

### 4.2. Interleukin 2 (IL-2)

Considering IL-2 as a potent Th1-type cytokine primarily synthesized by CD4+ T cells and, to a lesser extent, by CD8+ cells, NK cells, activated dendritic cells, and mast cells [85], studies on mice have revealed that the overexpression of IL-2 inhibits gestational viability [86]. In the human placenta, IL-2 is not detected at implantation sites [87], and women whose pregnancies end in miscarriage have significantly higher serum and endometrial levels of IL-2 [88,89]. In contrast to what is found in humans, the fetal porcine placenta has two peaks in IL-2 concentration: a smaller one at 30 dg and a larger one at 70 dg. These findings support the hypothesis that, at 30 dg, a controlled local proinflammatory environment must be generated at the fetomaternal interface due to the necessary placental remodeling coinciding with a period of extensive villi development [90] and the onset of embryonic ossification. A similar proinflammatory environment would also be required at 70 dg in porcine placenta to facilitate the necessary placental remodeling for sustaining the subsequent exponential fetal growth.

Regarding serum and endometrial levels of IL-2, contrary to what happens in human and murine gestation [91,92], it was observed that during porcine gestation, concentrations of IL-2 follow the pattern expressed by fetal placenta, albeit with lower values. It is worth noting that, during the term period, local and systemic concentrations reverse, meaning that in both the maternal and fetal placenta, the values decrease to baseline levels, while serum IL-2 concentrations increase significantly [51]. Based on these results, it has been suggested that the elevated serum levels of this cytokine at 114 days are necessary to trigger the molecular events that lead to labor and the expulsion of the placenta, as observed in a study of humans regarding the presence of various proinflammatory cytokines at the time of delivery [93].

### 4.3. Interleukin 4 (IL-4)

IL-4, also known as B-cell-stimulating factor, is a Th2 cytokine that exhibits pleiotropic effects during immune response. It is a glycoprotein secreted by CD4 Th2 lymphocytes, mast cells, and some CD8 cells. It has antitumor effects, inhibits the release of proinflammatory cytokines by activated monocytes, and regulates cytotoxic CD8 lymphocytes [94]. Cytokines associated with a Th2 response, such as IL-4, contribute towards embryo implantation, placental development, and fetal survival until the end of gestation in both humans and murine models [95].

Studies on human placenta suggest that IL-4 may be necessary during trophoblastic invasion [96], as only a weak expression of IL-4 has been found in trophoblasts during the first trimester, and its levels are virtually undetectable at term [97]. While placental IL-4 levels during the porcine implantation period have not been determined, IL-4 has been observed at the fetal–maternal interface during the latter stages of gestation. Its levels in the maternal placenta remained low and constant throughout pregnancy, but in the fetal placenta, they were elevated, particularly at 30 dg and 70 dg [51]. Meanwhile, Hanna et al. [97] observed that, in human maternal placenta, IL-4 was not detected. This suggests that fetal placenta may be the primary source of IL-4 found in pig placenta during gestation [51]. While controversies surround findings regarding IL-4 serum values during gestation in humans, with reported variations in its concentrations throughout pregnancy [94,97], porcine levels appear more consistent. Studies on pigs have shown increases in serum IL-4 levels during pregnancy, which in this case followed the patterns of expression generated by fetal placenta, peaking in concentration at 30 and 70 dg. It is worth noting that, similar to what occurs with IL-2 at term, the concentration of IL-4 in both placenta and serum reversed at this end stage of pregnancy, as it decreased back to basal levels in the placenta with a concomitant significant increase in serum expression [51]. IL-4 is known to be a potent inhibitor of Th1 lymphocytes, as it prevents the release of proinflammatory cytokines by activated monocytes and regulates cytotoxic CD8 lymphocytes [94]. Based on the findings regarding IL-4, it has been suggested that this cytokine must be present at the fetal–maternal interface (FMI) in order to regulate the proinflammatory environment established by the previously analyzed cytokines during periods of extensive placental remodeling. It has also been proposed that its elevated presence in serum at term is necessary for regulating the Th1 immune response generated at the time of birth, enabling the proper detachment of the placenta [51].

### 4.4. Interleukin 6 (IL-6)

IL-6 is a multifunctional cytokine that regulates various aspects of immune response in humans. This molecule is secreted by normal and transformed lymphoid and non-lymphoid cells, and its expression in monocytes is inhibited by IL-4, IL-10, and IL-13. IL-6 possesses both pro- and anti-inflammatory properties and is synthesized in response to certain microorganisms and other cytokines such as IL-1β [98].

The classification of IL-6 as an inducer of the Th1 or Th2 pathway has been controversial for several years. This controversy arises because the same molecule can exhibit both characteristics in human pregnancy depending on the dosage, the cellular source, and the gestational stage under study. Currently, it is considered that the presence of IL-6 shifts the Th1/Th2 balance towards a Th2 response. Towards the end of gestation, mRNA expression levels of IL-6 are four times higher than in the first trimester, possibly because it is involved in the proinflammatory process of pre-labor. During the first trimester of gestation, IL-6 has been implicated in the remodeling of placental tissues, as well as in the hematopoiesis and vascularization of placental villi [99].

Few studies have focused on an analysis of the physiological levels of IL-6 in sows during pregnancy. It has been reported that, during the first trimester of gestation, IL-6 plays a crucial role in various functions relating to placental development. Research by Koncurat et al. [61] on pigs has indicated its involvement in the remodeling of placental tissues and in hematopoiesis and the vascularization of placental villi. Some authors, such as Modric et al. [60], found an increase in the expression of the IL-6 gene on days 11/12 of conceptus development, as well as in the endometrium and placenta during the post-implantation phase (days 30 and 60 of gestation). In a more recent study, IL-6 concentrations significantly increased in homogenates from the porcine fetal placenta at 32 days of gestation, compared to the values found in the maternal placenta or serum during the different analyzed periods of 44, 50, 60, 70, 80 and 90 days of gestation [50]. These elevated levels of IL-6 in the fetal placenta compared to maternal placenta tissue and serum could be associated with an increase in asymmetric antibodies, as it may play a crucial role in the induction of the asymmetric glycosylation of IgG [100], which is crucial in preventing immune rejection of the fetus (see below; [62]). Furthermore, given the significantly higher fetal placenta IL-6 levels, which coincided with elevated E2 concentrations in the fetal placenta, it is important to note that the functions of this cytokine are not limited to the immune system and the inflammation process. Research has also demonstrated that it actively participates in fetal osteogenesis and angiogenesis processes [99,101]. Consistent with these studies, we have suggested that, during porcine gestation, the regulation of IL-6 may be influenced by estrogen concentration and could play a crucial role in the proper establishment of pregnancy, as well as in the initiation of fetal immune system development and ossification.

### 4.5. Interleukin 10 (IL-10)

IL-10, an immunosuppressive cytokine, generates immunotolerance, preventing the rejection of the fetal allograft by the maternal immune system [10]. In human and mouse pregnancy studies, it was assumed that the cellular source of IL-10 was either T lymphocytes or the trophoblast. However, a subpopulation of B lymphocytes, known as regulatory B10 cells, has emerged as a significant source of IL-10 [102]. The primary function of IL-10, produced by these B10 cells, is to maintain the delicate immunological balance required during pregnancy to establish fetal allograft tolerance. It has also been demonstrated that the IL-10 produced by B10 cells keeps dendritic cells in an immature state, thus inhibiting their antigen presentation capabilities and preventing the subsequent activation of T cells [103]. Additionally, it has been demonstrated that IL-10 exerts an inhibitory effect on uterine motility in late pregnancy to prevent premature labor.

Consistent with the findings made by Makris et al. [104], which revealed no correlation between placental IL-10 levels and serum levels in humans, our research group’s report similarly did not observe an association between these two concentrations in pigs [52]. This suggests that the placenta may not be the primary source of circulating IL-10 in pigs, in contrast to findings on the human placenta with high levels of this cytokine [97]. Elevated concentrations of circulating IL-10 have not been detected during porcine gestation. In one study, in both maternal and fetal porcine placenta tissue, the levels of this cytokine were nearly undetectable [52]. However, other authors found the expression of IL-10 and its receptor IL-10RA in the early-pregnancy *conceptus* and in the chorioallantoic membrane in pigs during middle–late gestation. In the endometrium, IL-10 mRNA expression was found to be higher at 15 dg but to decrease until day 90, at which point it maintained a constant concentration [15]. The difference between the two studies could be related to methodology differences. Han et al. [15] used RT-PCR to determine mRNA and immunohistochemistry, while Velez et al. [52] used ELISA. Previous to these contradictory results, IL-10 mRNA expression in pigs had only been found in term placenta [105] and in 30 dg placenta.

In pregnant pig serum, three significant peaks in IL-10 concentration were observed at 17, 60, and 114 dg [52]. This work supported the earlier theory proposed by Jensen et al. [103] following their murine study, indicating that the high serum concentration of IL-10 found in pigs at days 17 and 60 were likely explained by its regulatory role, and that it may serve to safeguard the mother systemically from the local proinflammatory profile generated both at the time of implantation and at 60 and 70 dg. The high serum concentration at the time of delivery may suggest that, in pigs, this cytokine could be associated with an inhibitory effect to prevent excessive tissue damage during this event, as postulated by Van Engelen et al. [106], in their cattle study.

### 4.6. Interleukin 12 (IL-12)

IL-12 is a cytokine that plays a significant role in inducing cell-mediated immune responses and enhancing the activity of cytotoxic T lymphocytes and natural killer cells. It is also involved in the differentiation of naïve T cells into the Th1 subset [107]. This cytokine induces the synthesis of IFN-gamma (IFN-γ) in human peripheral blood mononuclear cells in vitro, and macrophages and dendritic cells are the primary sources of IL-12 in many tissues.

There have been very few studies that have assessed the presence and role(s) of IL-12 during porcine gestation. Williamson et al. [63] detected concentration peaks at 70 and 90 dg in serum. Notably, these were lower than the values found in maternal placental homogenates, which remained high and constant throughout pregnancy. In contrast, in fetal placental homogenates, a concentration peak was found only during the 32–44 dg period, and these levels were higher than that determined in maternal placental homogenates. Han et al. [15] found a high expression of IL-12 only in the pre-implantational stage; however, these authors did not analyze placentas at 32–44 dg.

It is known that a classical role of IL-12 is to promote the Th1-type response, activating macrophages and NK cells and inducing the production of IFN-γ, thereby enhancing the cytolytic activity of T lymphocytes and NK cells. It is also understood that IFN-γ increases the production of IL-12 and, in turn, that IL-12 can act synergistically with IL-18 to trigger a Th1 response [108]. Based on the background of this molecule and the limited research reported in pigs, it is hypothesized that IL-12, specifically during a period of porcine gestation, may participate in the molecular dialogue established at the fetal–maternal interface, helping to regulate the proinflammatory environment that occurs at particular times throughout pregnancy in sows.

### 4.7. Interleukin 15 (IL-15)

IL-15 plays a crucial role in the differentiation of uNK cells in mice, and previous studies have demonstrated that its endometrial expression in humans and mice is strictly regulated by progesterone (P4) [109]. Research on humans has revealed the presence of IL-15 and its mRNA in the non-pregnant endometrium, decidua, and placenta [110], and has demonstrated its role in influencing uNK cytotoxicity and proliferation [87]. In all eutherians studied, including pigs, this cytokine is produced in the uterus in response to the transcription factor HAND-2, an important regulator of implantation and a key component of pregnancy conservation [14].

Few studies have investigated the presence of IL-15 during gestation; they mainly focus on the first trimester of human pregnancy, a species with hemochorial and deciduous placentation [87,111,112]. To date, research on IL-15 in sows during gestation reveals that non-pregnant sows exhibit a higher expression of IL-15 in uterine homogenates that significantly differs from the values found during gestation. In contrast, the serum concentration of IL-15 in pregnant sows was higher and displayed a pulsatile pattern throughout pregnancy, except at 70 days when it decreased significantly. During this period, there was an increase in IL-15 in both the maternal and fetal porcine placental components relative to serum values, suggesting that IL-15 may be necessary at the fetal–maternal interface during that gestational period [63].

While IL-15 in women has been observed to be related to the early appearance of NK cells in the pregnant endometrium, reports from Williamson et al. [63] do not support this observation, as increases in IL-15 in porcine placental components occurred only at 60–70 dg in their research. Although it is known that NK cells appear early in the pregnant porcine endometrium [11,18,25], this cytokine must serve different functions during human and porcine pregnancy. In humans, it has been suggested that IL-15 mRNA expression is regulated by steroid hormones, especially P4, as IL-15 has been found in the culture supernatant of decidual cells, and the addition of P4 stimulated secretion [113].

Consistent with this research, in pigs, elevated concentrations of IL-15 and P4 were found in serum at 40 days of pregnancy, and the same occurred at 60 and 70 days when levels also significantly increased in fetal placental components [63]. Cristofolini et al. [84] reported the presence of abundant phagocytic cells in the porcine endometrium at 60 days of pregnancy, leading us to consider that the IL-15 detected in the fetal placental extracts during that gestational period could be due to the presence of P4, which stimulates the biosynthesis of IL-15. These results suggest that while the functions of IL-15 may differ among species, the mechanisms that regulate its expression could be similar.

### 4.8. Interleukin 18 (IL-18)

IL-18 is a protein that promotes a Th1 response. It has been reported that its expression during the secretory endometrial phase can modulate the Th1/Th2 cytokine network during embryonic implantation [114]. Similar to IL-15, IL-18 is also produced, among other cells, by macrophages, and promotes the production of IFN-γ and enhances the activity of NK cells, inducing a Th1-type immune response [115].

Studies have shown that, in non-pregnant females, the concentration of IL-18 is higher in uterine extracts than in serum from both non-pregnant and pregnant sows. Furthermore, it has been shown that IL-18 is expressed in pulsatile peaks only in fetal placental extracts throughout the course of gestation [63]. According to Ashworth et al. [116], IL-18 is produced by the endometrium and is associated with porcine implantation, as it is linked to the peak of IFN-γ expression observed between days 15 and 18 of gestation. However, Willamson et al. [63] did not find this association in the earlier stages of pregnancy, but instead observed IL-18 later from day 44 of pregnancy in fetal placental extracts, as well as at days 65 and 70, while the presence of IFN-γ was notable in their research on days 17 and 32.

### 4.9. Interleukin 23 (IL-23)

IL-23 is a cytokine proinflammatory that stimulates Th17 cells. In pigs, this cytokine was present in the uterus but only some hours after mating, likely in relation to the inflammatory reactions that occur in this moment. Therefore, it is related to infection prevention [64]. No other reports relating to this cytokine were found relating to pig gestation; therefore, little is known about other potential expression patterns and functions.

### 4.10. Interferon Gamma (IFN-γ)

IFN-γ is known as a proinflammatory cytokine of the Th1 type, primarily produced by stimulated lymphocytes and, in the case of pigs, it is also synthesized by trophoblast cells [65]. IFN-γ expression has been associated with embryonic and fetal death in various species [117]. However, recent research has highlighted its relevance in the early stages of human gestation, where its essential role in normal implantation has been demonstrated [118].

In previous studies, several researchers have reported on the high expression of IFN-γ in porcine uterus during the pre-implantation period [44,119,120]. It also has angiogenic functions in the porcine placenta [27]. This increase in IFN-γ has been associated with the characteristic conceptus loss that occurs around day 20 of gestation, but notably not with the loss that occurs on day 50 [4]. The concentration of this cytokine in sow placenta has been observed to rise at 17 dg and then remain at basal levels in the later stages of pregnancy, both in maternal and fetal placental tissue. These low values persist throughout the remainder of gestation [52]. Therefore, it has been reported that IFN-γ could be maintained in the uterine compartment and act as a direct effector in the depolarization of the apical membrane of the endometrial epithelium, leading to endometrial remodeling, a crucial process for successful embryonic implantation [118].

In contrast to fetal and maternal placenta, at a systemic level, significant increases in IFN-γ levels within the serum have been found at 60 dg [52], coinciding with the period when high serum levels of IL-1β have previously been reported [51]. This notable increase in IFN-γ in serum at 60 days could establish a pattern of proinflammatory cytokines that favor the characteristic placental remodeling of this particular stage of porcine gestation. Findings suggest that the source of IFN-γ in this context may not be placental, pointing to the involvement of another organ(s), as elevated concentrations of IFN-γ have been observed in serum but not in the placenta itself [52]. Furthermore, it is relevant to mention that, although the increase in IFN-γ is evident in the late hemochorial placenta and is related to the necessary changes required for parturition in some species [93,121], this does not appear to occur in pigs, a species with an epitheliochorial placenta. In a previous report, it was noted that IL-1β and IL-2 increased in serum but not at the placental interface during term pregnancy [51]; based on these results, it is likely that other proinflammatory cytokines take on the roles of IFN-γ during pig parturition.

### 4.11. Tumor Necrosis Factor Alpha (TNF-α)

TNF-α is a pleiotropic multifunctional polypeptide [66] can be synthesized by various cell types [122], including those of the female reproductive system [123]. TNF-α is another cytokine involved in the early stages of porcine gestation [43,70]. Peak TNF-α expression has been detected in both the endometrium at 15 days and the corpus luteum at 17 days [66,69]. In the early stages of pregnancy, during implantation, TNF- α regulates the expression of progesterone [67,71] and E2 [68]. It is also involved in cell differentiation, tissue remodeling, and apoptosis in the early phase of porcine gestation [69]. So far, the role of TNF-α in late gestation remains unknown [49], but it has been demonstrated that this molecule has a detrimental effect on embryonic development in rats, mice, and humans due to its ability to activate both proapoptotic and antiapoptotic signaling cascades [49,66,124]. Therefore, it is understood that the expression of TNF-α could be present at low levels, or absent, during the latter stages of pregnancy in pigs [105].

### 4.12. Leukemia Inhibitory Factor (LIF)

LIF is a cytokine that exhibits a variety of functions, including cell proliferation, differentiation, and survival, which are all important for embryo development and implantation [72]. Studies on humans and mice have reported that this cytokine is expressed in the endometrium during the implantation window [125], promoting interactions between trophoblastic cells and decidual leukocytes [126,127].

In pigs, LIF is secreted by the conceptus and the endometrium between days 10 and 14 of gestation, suggesting that in this species with adequate placentation, it also plays a crucial role during implantation [60,72,73]. Furthermore, Yoo et al. [128] demonstrated that LIF expression increased in the endometrium during the late diestrus phase of the estrous cycle, in addition to during the middle and end of pregnancy. Similar to IL-6, LIF expression is controlled by IL-1 and estrogen [129]. As described by Blitek et al. [72], the highest concentrations of LIF protein have been detected following uterine flushes of pigs on day 12 of gestation, and are therefore temporally associated with the expression of conceptus IL-1β2 and estrogen synthesis during maternal recognition of pregnancy. Recently, Cambra et al. [130] found mRNA and the protein expression of LIF and its receptor in the endometrium of pig placenta during the implantational and post-implantational periods (18 and 24 dg).

### 4.13. Transforming Growth Factor Beta (TGFß)

As with other cytokines such as IL-6 and IL-10, the Th3 cytokine TGF-β1 has been shown to be expressed on the surface and in the glandular epithelia of the porcine endometrium after insemination, in particular [75]. Subsequently, mRNA levels of TGF-β1 were evident and increased between 10 and 15 dg in the endometrium and conceptus [74]. We have not found any references for this cytokine relating to its expression or function in more advanced pregnancy stages.

## 5. Humoral Immunity in Pregnancy

### 5.1. Asymmetric IgG Antibodies

During pregnancy, the predominance of the immunoregulatory Th2/Th3/Tr1 response leads to an increased humoral immune response. B cells with different functions have also been identified in human and mouse placentas. In humans, a subtype of B cells, Breg, principally produce IL-10, resulting in anti-inflammatory activity. In mice, B1 and B2 cells have been identified during gestation. Importantly, two types of antibodies were secreted during human gestation: autoantibodies were involved in pathological processes such as preeclampsia, and asymmetric antibodies with a protective role were detected [131]. Similar B-cell subtypes have not yet been reported in pig placenta, but have been described in other organs and in the circulation of this species [132]. The use of the terms Th3 and Tr1 cytokines derive from other species and not are related to the cell types that produce in pig the cytokines.

The production of blocking or asymmetric antibodies is crucial for establishing pregnancy. Antibodies of the IgG isotype with the same subtypes as the precipitating antibodies exhibit different immunological and biological behavior [133]. Asymmetric IgG antibodies do not form aggregates with the antigen, and consequently, they do not activate effector biological mechanisms such as complement lysis, phagocytosis, or in vivo antigen clearance. They do not precipitate the antigen because only one of the two antigen-binding regions in the Fab fragment contains a mannose-rich hydrocarbon residue, which creates a steric hindrance and a marked decrease in antigen affinity [100,133].

Studies on pregnancy regulation in humans and mice have observed an increase in asymmetric IgG antibodies in serum and placental extracts [134]. In normal immune responses, approximately 15% of functional asymmetric IgG antibodies have been observed in mammalian sera, while these values increased to 30% in human and murine sera during pregnancy [135]. The presence of these asymmetric antibodies has also been demonstrated in rats, sheep, rabbits, horses, cows, and in donkeys. In preliminary studies on pigs, the presence of asymmetric IgG antibodies has also been determined in porcine sera and in placental extracts from both maternal and fetal tissues [62,136].

In pregnant people and mice, alongside the increase in asymmetric IgG antibodies in serum and placental extracts [134], research has also reported that both IL-6, as well as estrogens and progesterone, influence the synthesis and glycosylation of asymmetric IgG antibodies [137,138,139,140,141,142]. The function of the asymmetric IgG antibodies is believed to be related to the protection of the fetus from maternal immune attack. Williamson et al. [50] and Koncurat et al. [61] detected the presence of IL-6 during early porcine pregnancy (around 32 dg) as located solely in fetal placental extracts. This interleukin, which promotes IgG synthesis, is necessary for the protection of the conceptus, since IgG and its receptor have been found in histological sections of both maternal and term fetal placenta [143,144].

Recent studies indicate that there are no significant differences in the percentage of asymmetric IgG antibodies versus symmetric antibodies in serum between non-gestational and gestational sows overall; however, there were differences in the percentage of asymmetric IgG antibodies among the different gestational periods studied [62]. This difference in the percentage of symmetric and asymmetric IgG antibodies found in serum across different gestational periods could be one of the factors regulating maternal immune response. A decrease in serum asymmetric IgG antibodies was only observed at 30 dg, whereas in late gestation, increases were found. The serum decrease in asymmetric IgG antibodies at the beginning of pregnancy is likely due to the transfer of these antibodies to the fetal–maternal interface in order to preserve the fetal allograft. Meanwhile, the increase in serum asymmetric IgG antibodies at the end of gestation may reflect a compensatory mechanism to offset the sharp drop in IgG concentration in serum when most IgGs are concentrated in the mammary glands destined to be part of the colostrum [145,146].

The increase in asymmetric IgG antibodies recorded at 65–70 dg in maternal placental homogenates coincides with the period when the placenta stops increasing in weight and volume, reaching a plateau in porcine pregnancy [147,148,149]. This suggests that IgG antibodies synthesized by the mother are concentrated in the maternal placenta, particularly at the fetal–maternal interface, to protect the fetus from the maternal immune system. While the placenta does not then increase in weight or volume, it continues to undergo a cellular remodeling of its structure, particularly of the villi that make up the interface, via apoptotic mechanisms [84,150]. Thus, the presence of IgG in the new cells exposed at the interface is necessary to prevent rejection. The percentage of asymmetric IgG antibodies significantly decreases at term in maternal placental homogenates [151]. This could be related to the aforementioned concentrations in the serum and mammary glands. Regarding fetal placental homogenates, it is noteworthy that high percentages of asymmetric IgG antibodies were detected in these extracts across all of the gestational periods studied. This is intriguing because the porcine fetus only begins to synthesize its first antibodies at around 50 dg [152,153,154]. Therefore, it is hypothesized that these asymmetric antibodies must have been produced by the mother.

It is also reasonable to assume that the IgG antibodies found in fetal and maternal placental extracts are predominantly of the asymmetric IgG type, intended to protect the fetal allograft. Indeed, if they were symmetric IgG antibodies, they would have the capacity to trigger tissue destruction. Therefore, it is estimated that the majority of the detected IgG antibodies are asymmetric IgG antibodies synthesized by the mother’s B lymphocytes, and there is likely a special type of transport through the luminal uterine epithelium into the fetal placenta. High levels of asymmetric IgG antibodies were only found in fetal placental extracts, and these levels remained constant throughout pregnancy and were detected from 30 dg onwards.

### 5.2. Fc Gamma Receptor

In order for IgG antibodies to traverse through tissues, they need to be bound to their receptor, particularly the neonatal Fc receptor (FcRn) [155]. Thus, FcRn shields IgG from catabolism and enables the maintenance of constant antibody levels in the serum. FcRn receptors are primarily found in vascular endothelial cells and are recycled internally to prevent degradation. They are also expressed in other tissues and organs, such as bone marrow, antigen-presenting cells, the liver, mammary glands, and intestine, and it has been suggested that they modulate the transport of IgG through tissues to reach their sites of action [156].

In porcine gestation, a positive expression of the IgG Fc receptor has been observed in histological sections of both maternal and fetal placentas at 30 days of pregnancy [144]. Only the presence of FcRn allows the transcytosis of IgG across epithelial barriers, and this mechanism helps explain the presence of IgG detected by immunohistochemistry tests on histological sections during periods when the fetus has not yet acquired the ability to synthesize IgG. During porcine pregnancy, the presence of IgG molecules and their Fc receptor at the fetomaternal interface from 30 dg—particularly asymmetric IgG, which would arrive via transcytosis from the maternal epithelium—may play a role in the immune protection of the fetus. This highlights the role of the humoral immune system during porcine gestation.

Based on the reported findings, it has been postulated that one mechanism through which the sow protects her embryos/fetuses includes IgG transcytosis [157,158], which occurs through the maternal placenta. It is believed that IgG molecules become trapped at the fetomaternal interface in the space between the luminal endometrial epithelium and the trophoblast. This mechanism would explain the immunoglobulin observed through immunohistochemistry tests on histological sections of fetal placenta in individuals that have not yet acquired the ability to synthesize IgG molecules.

## 6. Extracellular Vesicles and Mirna in Pig Gestation

Small RNAs, such as miRNAs, have a principal function in regulating immune responses, and are generated by the post-translational inhibition of gene expression. Some of these RNAs are found in secreted extracellular vesicles (EVs) and have been associated with embryonic loss and immune placental regulation and have been studied in diverse species, including bovines [159] and humans [160]. Related to the importance of molecular communication between the uterus and the embryo in pigs, there are reports that focus on studying the roles of EVs (including exosomes and microvesicles) and on understanding how research into this area can contribute towards improving pig reproduction, helping us gain a better comprehension of the challenges related to gestation and embryonic implantation. Hua et al. [161] focused on studying the EVs present in uterine flushing fluids from pigs. These EVs contain small RNA molecules that play an important role in embryonic implantation. The study described, identified, and characterized different types of RNAs in EVs during different stages of gestation and revealed how some of these RNAs were related to important processes such as immunization, endometrial receptivity, and embryonic development. Hua et al. [162] focused on the importance of interactions between signaling molecules in the uterus and the porcine conceptus for successful implantation. They described how EVs in the uterine luminal fluid play a role in conceptus development and how these EVs are primarily derived from endometrial epithelial cells. Meanwhile, Bidarimath et al. [163] highlighted the role of EVs, such as exosomes, in bidirectional communication between fetal and maternal cells during gestation. They highlighted the unique character of porcine placentation due to the importance of the trophoblast in the recruitment of immune cells in the uterus and showed that extracellular vesicles could be of great importance in this process. miRNAs were found in porcine trophectoderm cells derived from day 12 of pregnancy, and in porcine endothelial cells, as well as exosomes released by both [164]. In a more recent study, Hua et al. [161] reported changes in the transcriptome of trophoblastic and endometrial cells generated by the EVs found in the uterine fluids of pig in the implantational period. Amongst the genes with modified expression were some of the interleukins (known to be important for pig gestation regulation), their receptors, and chemokines involved in immune cell migration [161]. Based on this background knowledge, it is essential to gain a deeper understanding of communication between the endometrium and the embryo/fetus in pigs during both the implantation period and throughout the rest of gestation. In other species, such as in humans, there are numerous studies that analyze the effects of these EVs on immune pregnancy regulation. Currently, there is also an increasing number of studies relating to the function of extracellular vesicles as mediators of this communication. Studying these areas will improve our understanding of biological challenges in successful reproduction [165].

## 7. Conclusions: Immune Response during Different Pig Pregnancy Periods

In the four-day period following mating, there is an increase in proinflammatory factors and cells related to the increase in serum estrogen, with an infiltration of immune cells including neutrophils, macrophages, dendritic cells, and T lymphocytes. In the pre-implantation stage (5–12 days) there is a decrease in both estrogen and proinflammatory factors, and an increase in progesterone levels [166]. In relation to the immune cells, there is an increase in dendritic cells and macrophages [167]. The implantational period between days 12 and 25–28 [168] could be divided into different stages within the initial implantational stage. The production of estrogen for the conceptus starts an inflammatory response with increase in NK cells and some proinflammatory cytokines such as IL-B1 and 2. When the embryos attach, other proinflammatory cytokines including IL-18, IFN-γ, and TNF-α increase. Once implantation occurs at 25 dg, there is a concomitant increase in anti-inflammatory cytokines such as IL-4, IL-6, and IL-10, alongside the related endometrial vascular remodeling and placentation angiogenesis initiation [166].

In most mammalian species studied, a long period with high levels of Th2 anti-inflammatory cytokines occurs from the end of implantation to peripartum. However, as was revealed in the previous section, in pigs there are gestational days where there are increases in diverse cytokines including proinflammatory Th1 ones. At some gestational points where these cytokines have differential expression, some important events occur. For example, between 60 and 90 dg, changes in cell death processes happen [169], differential expression also occurs during angiogenesis [170], and changes in adhesion molecule expression have also been found [171,172,173].

Work on the prepartum pig [51,52] has also demonstrated increases in serum, but not in placental proinflammatory cytokines, as is described in other species [106,174,175,176,177]. Indeed, in pigs, serum levels of IL-1β remained at basal levels during pregnancy but increased significantly at the end of pregnancy, likely with the sole purpose of activating the proinflammatory immune system to prepare for placental expulsion during labor [51,52].

## 8. Future Perspectives

The importance of embryo death in pigs has resulted in many studies analyzing the gestational and fetal–mother interface immunology of this species. In particular, the changes in cytokines and immune cells throughout the middle and late gestational stages, including peripartum, have been studied in depth. These works have shown that specific important aspects, such as the importance of asymmetric antibodies, must be studied further.

Despite the large advances made to date, continued research is required in relation to studying the molecules involved in the formation of the non-invasive fetomaternal interface in porcine species in order to further investigate their regulation. The ultimate goal of this work is to develop strategies that can restore immunological balance in sows with a high rate of early embryonic reabsorption or fetal loss. Research in this field and improved management strategies will lead to significant advancements in improving the productivity of this species, with a positive impact on the economy of pork farming companies and food security.

## Figures and Tables

**Table 1:** Cytokines in pig placenta.

Molecules	General Considerations	Pig	References
Cytokines			
IL-1β	A proinflammatory cytokine that acts as a central mediator of inflammation and innate immunity in mammals.	The porcine conceptus produces IL-1β during early development and elongation and is necessary for this process. This cytokine increases permeability of endometrial blood vessels, facilitating fetal–maternal hemotrophic exchange. Until day 14, the inhibition of this cytokine generates a failure in conceptus elongation. IL-1β concentration shows a peak within the fetal placenta at day 60 and in the maternal placenta at day 70, and decreases at day 114.	[51,53,54,55,56,57,58,59]
IL-2	A potent Th1- cytokine, primarily synthesized by diverse cell types.	The porcine placenta has two peaks in IL-2 concentration: a smaller one at 30 dg and a larger one at 70 dg. In both the maternal and fetal placenta, the values decrease to baseline levels, while serum IL-2 concentrations increase significantly. It has been suggested that the elevated serum levels of this cytokine at 114 days are necessary to trigger the molecular events that lead to labor and the expulsion of the placenta.	[51]
IL-4	Also known as B-cell-stimulating factor, this is a Th2 cytokine that has pleiotropic effects during immune response. It is secreted by CD4 Th2 lymphocytes, mast cells, and some CD8 cells.	Its levels in the maternal placenta remain low and constant throughout pregnancy, but they are elevated in the fetal placenta, particularly at 30 dg and 70 dg. Serum IL-4 levels peak in concentration at 30 and 70 dg. At term, it decreases back to basal levels in the placenta with a concomitant significant increase in serum.	[51]
IL-6	Regulates various aspects of the immune response. It is considered that IL-6 shifts the Th1/Th2 balance towards a Th2 response.	IL-6 gene expression increases on days 11/12 of conceptus development and in the endometrium and fetal placenta during the post-implantation phase (days 30 and 60 of gestation). Concentration is higher at 32 gd in the fetal placenta.	[60,61,62]
IL-10	An immunosuppressive cytokine that generates immunotolerance, preventing the rejection of the fetal allograft by the maternal immune system.	In both maternal and fetal porcine placenta tissue, the levels of this cytokine were nearly undetectable. In pregnant pig serum, three significant peaks in IL-10 concentration were observed at 17, 60, and 114 dg.	[52]
IL-12	Enhances the activity of cytotoxic T lymphocytes and NK cells, is involved in the differentiation of naïve T cells into the Th1 subset, and induces the synthesis of IFN-gamma.	IIL-12 expression in the pregnant uterus was high in the pre-implantational stage. Concentration peaks of IL-12 at 70 and 90 dg were detected in serum. In maternal placenta, the concentration remained high and constant throughout the pregnancy. In fetal placental homogenates, a concentration peak was found only during the 32–44 dg period.	[15,61]
IL-15	Influences uNK cytotoxicity and proliferation.	The serum concentration of IL-15 in pregnant sows was high, except at 70 days when it decreased significantly. At 70 dg, there was an increase in maternal and fetal porcine placental components.	[63]
IL-18	Induces a Th1 response.	IL-18 is expressed in fetal but not in maternal placenta throughout the gestation period.	[63]
IL-23	A cytokine proinflammatory that stimulates Th17 cells.	IL-23 is present in the uterus but only some hours after mating, and is found in relation to the inflammatory reactions that occur in this moment.	[64]
IFN-γ	A proinflammatory cytokine Th1, primarily produced by stimulated lymphocytes.	In pigs this is also secreted by trophoblast cells. The concentration of this cytokine rises at 17 dg and then remains at basal levels in the later stages of pregnancy, both in maternal and fetal placenta. In serum, a significant increase in IFN-γ levels was found at 60 dg.	[52,65]
TNF- α	A pleiotropic proinflammatory cytokine synthesized by diverse cell populations.	TNF-α is involved in the early stages of porcine gestation in regulating the expression of progesterone and estrogens. Peak TNF-α expression was found in the endometrium at 15 gd. It is involved in cell differentiation, tissue remodeling, and apoptosis in the early phase of porcine gestation.	[43,66,67,68,69,70,71]
LIF	Regulates cell proliferation, differentiation, and survival during embryo development and implantation.	LIF is secreted by the conceptus and the endometrium between days 10 and 14 of gestation, suggesting a crucial role during implantation. Some authors found mRNA and protein expression of LIF and its receptor in the endometrium of pig placenta during the implantational and post-implantational periods (18 and 24 dg).	[60,72,73]
TGF-β1	A pleiotropic growth factor and an immunomodulatory cytokine.	TGF-β1 is expressed in the surface and glandular epithelia of porcine endometrium after insemination. mRNA TGF-β1 increased between 10 and 15 dg in the endometrium and conceptus.	[74,75]

**Table 2:** Cytokines in different gestational periods and their principal events.

Gestational Period	Principal Local Events	Predominant Cytokines
Four days post mating	Proinflammatory stages. Estrogen predominant hormone. Infiltration of neutrophils, dendritic cells, macrophages, and T cells.	Proinflammatory
Pre-implantation (5–12 gd)	Increases progesterone and decreases estrogen. Anti-inflammatory stages. Increases macrophages.	Decreases proinflammatory and increases anti-inflammatory effects.
Implantational stage (12–25 gd)	Conceptus production of estrogen with increases in NK cells.	Initially, Il-ß1 and 2 are the most important. Some days later, other proinflammatory cytokines such as IL-18, IFN-γ, and TNF-α increase.
Final implantational stage (25–28 days)	There is a remodeling of uterine vasculature and angiogenesis begins in embryonic placenta.	Increases in anti-inflammatory cytokines such as IL-4, IL-6 and IL-10.
Rest of the pregnancy (except for 60–90 dg)	Placentation is established. The conceptus grows.	Th2 anti-inflammatory cytokines are predominant,
60–90 gd	Placenta remodeling with cell death, angiogenesis, and changes in adhesion molecules.	Increases in diverse cytokines, including some Th1.
Peripartum (114 gd)	Inflammatory response to eliminate conceptus.	There is not an increase in local Th1 cytokine, as in other mammals; however, some proinflammatory ones such as IL-ß1 increase in serum.

## Data Availability

Data contained within this article.
